# First Autochthonous West Nile Lineage 2 and Usutu Virus Infections in Humans, July to October 2018, Czech Republic

**DOI:** 10.3390/pathogens10060651

**Published:** 2021-05-24

**Authors:** Hana Zelená, Jana Kleinerová, Silvie Šikutová, Petra Straková, Hana Kocourková, Roman Stebel, Petr Husa, Petr Husa, Eva Tesařová, Hana Lejdarová, Oldřich Šebesta, Peter Juráš, Renata Ciupek, Jakub Mrázek, Ivo Rudolf

**Affiliations:** 1 Public Health Institute, Partyzánské nám. 7, 702 00 Ostrava, Czech Republic; hana.zelena@zuova.cz (H.Z.); jakub.mrazek@zuova.cz (J.M.); 2 Department of Biomedical Sciences, Faculty of Medicine, University of Ostrava, Syllabova 19, 703 00 Ostrava, Czech Republic; 3 Department of Infectious Diseases, Hospital Břeclav, U Nemocnice 3066/1, 690 74 Břeclav, Czech Republic; jana.kleinerova@post.cz; 4 Institute of Vertebrate Biology, The Czech Academy of Sciences, Květná 8, 603 65 Brno, Czech Republic; sikutova@ivb.cz (S.Š.); strakova.p@centrum.cz (P.S.); 5 Veterinary Research Institute, Hudcova 70, 621 00 Brno, Czech Republic; 6 Department of Infectious Diseases, University Hospital Brno, Jihlavská 20, 602 00 Brno, Czech Republic; kocourkova.hana@fnbrno.cz (H.K.); stebel.roman@fnbrno.cz (R.S.); husa.petr@fnbrno.cz (P.H.); husa.petr2@fnbrno.cz (P.H.J.); 7 Department of Infectious Diseases, Faculty of Medicine, Masaryk University, Kamenice 753/5, 625 00 Brno, Czech Republic; 8 Department of Health Insurance, University Hospital Brno, Jihlavská 20, 602 00 Brno, Czech Republic; tesarova.eva@fnbrno.cz; 9 Transfusion and Tissue Department, University Hospital Brno, Jihlavská 20, 602 00 Brno, Czech Republic; lejdarova.hana@fnbrno.cz; 10 Regional Public Health Authority of the Southern Moravia Region, Jeřábkova 4, 602 00 Brno, Czech Republic; oldrich.sebesta@tiscali.cz (O.Š.); peter.juras@khsbrno.cz (P.J.); renata.ciupek@khsbrno.cz (R.C.)

**Keywords:** West Nile virus, Usutu virus, mosquito-borne infections, human

## Abstract

We present epidemiological, clinical and laboratory findings of five Czech patients diagnosed with autochthonous mosquito-borne disease—four patients with confirmed West Nile virus (WNV) and one patient with Usutu virus (USUV) infections, from July to October 2018, including one fatal case due to WNV. This is the first documented human outbreak caused by WNV lineage 2 in the Czech Republic and the first record of a neuroinvasive human disease caused by USUV, which illustrates the simultaneous circulation of WNV and USUV in the country.

## 1. Introduction

West Nile virus (WNV) is a mosquito-borne arbovirus belonging to the family *Flaviviridae*, genus *Flavivirus*, Japanese encephalitis serocomplex. In nature, it circulates mainly among birds and mosquitoes of genera *Culex*, *Anopheles*, *Culiseta*, *Uranotaenia* or *Coquilletidia*. WNV is a causative agent of West Nile fever, a mosquito-borne disease affecting horses and humans, the latter serving as so-called dead-end hosts [1]. Since 2004, highly virulent WNV lineage 2 has appeared in Europe, causing sporadic outbreaks in Hungary (2008), Greece (2010) and Serbia (2012) [2]. In the Czech Republic, WNV research in birds, mosquito vectors and humans has a long tradition. Importantly, highly virulent WNV lineage 2 (WNV-2) strains have been repeatedly documented in *Cx. modestus* populations on local fishponds [3,4]. Interestingly, no case of WNV-2 infection in humans had been documented before the 2018 season.

Usutu virus is a mosquito-borne flavivirus (family *Flaviviridae*, genus *Flavivirus*), in the Japanese encephalitis virus serocomplex. Like WNV, it circulates among birds and ornithophilic mosquitoes, with demonstrated pathogenicity to a wide variety of wild and domestic birds. It emerged in 2001 in Austria with the highest mortality recorded in blackbirds [5]. Since then USUV has spread further to central and western European countries including the Czech Republic, where it has established itself among blackbirds [6,7] and mosquitoes [8]. Since its introduction in Europe, there have been several reports of neuroinvasive disease in immunocompromised [9] and immunocompetent humans [10,11]. Similar to WNV, USUV has been detected in asymptomatic blood donors in Italy [12], Austria [13] and Germany [14].

The aim of this report was to summarize the epidemiological and clinical characteristics of patients diagnosed by infection with either WNV-2 or USUV in the Czech Republic and to expand our knowledge of these emerging mosquito-borne diseases in Central Europe.

## 2. Case Reports

We summarized epidemiological, clinical and laboratory findings collected from five patients diagnosed with WNV or USUV infections. Specific data are summarized in Table 1, Table 2 and Table 3. All but one had no history of traveling to a known WNV endemic area. One patient (Case 1) reported previous vaccination against tick-borne encephalitis virus (TBEV), a related tick-borne flavivirus circulating in the area, that might present with cross-reactive immunity, which could subsequently complicate an accurate final diagnostic result. All patients reside in South Moravia.

### 2.1. Description of Cases

#### 2.1.1. Case 1

On 3 August 2018, a 74-year-old man was examined at the Infectious Diseases Clinic of Brno University Hospital for fever up to 40 °C for 12 days. Fever was accompanied by flu-like symptoms and severe headaches. The patient reported diarrhea during the first 4 days and observed macular rash on the skin on the 6th day after the onset of the fever. Apart from dehydration with borderline hypotension, the clinical examination revealed no obvious pathology. The patient had no symptoms of meningitis; no cerebrospinal fluid was tested. Basic laboratory samples were taken as early as 31 July 2018 (see Table 2). No increase in inflammatory parameters, no leukocytosis and no other pathology was found. Treatment was symptomatic with antipyretics, analgesics and hydration by infusion; there was no antibiotic therapy. A clinical follow-up was performed after 4 days, rash and fever subsided and temperatures also decreased after 13 days. Exhaustion and occasional headache persisted. Further stool and urine cultures were negative, as were CMV serology and stool virology. Inflammatory parameters remained low. In order to exclude infectious foci, a number of additional examinations were performed (chest X-ray, abdominal and intestinal ultrasound, dentistry, echocardiography). The only significant finding was tooth decay; the dentist recommended the extraction of several teeth over time, but ruled out the odontogenic etiology of the fever. The patient’s exhaustion and fatigue slowly disappeared, the fever did not return, and recovery lasted approximately 7 weeks. The man was not hospitalized; he was in the home care of his wife—a doctor. The patient gradually returned to regular activities, including recreational sport. The diagnosis of West Nile Fever was made ex post. The serological finding of WNV correlated with the protracted clinical course of the febrile viral disease.

**Epidemiological background:** The patient’s place of residence is metropolitan Brno. From June 2018 the patient slept outside on the covered terrace of a family villa. The patient stayed in Brno—except for a day trip on 6 May 2018 to the aqua park in Laa an der Thaya (Austria). From 31 May 2018 to 20 July 2018 the patient spent a total of 10 days in a cottage by a pond north of Brno. The patient was vaccinated against TBEV.

#### 2.1.2. Case 2

The second case report concerns a 72-year-old female patient admitted on 13 August 2018 at the internal department of the Břeclav Hospital for general weakness, diarrhea and myalgia lasting 6 days. She was a polymorbid patient treated for rheumatoid arthritis, lichen planus, chronic gastritis and thyroiditis. On the day of admission, the patient was found at home and could not move. She had the status of odontogenic etiology of fever in the internal medicine department. The patient gradually deteriorated, and a quantitative impairment of consciousness appeared on the 6th day of hospitalization (18 August 2018). The patient was transported to the department of anesthesiology, intensive care medicine and resuscitation where she was promptly intubated, artificial sleep was induced and artificial lung ventilation was started. Computed tomography (CT) of the brain showed non-specific hypodense areas in brain tissue. Furthermore, lumbar puncture was supplemented as part of differential diagnosis of the disturbance of consciousness. Biochemical and cytological findings in cerebrospinal fluid corresponded to aseptic neuroinfection. Serology showed borderline IgM and weakly positive IgG antibodies against tick-borne meningoencephalitis in both blood and cerebrospinal fluid. Despite the established intensive therapy, the patient further deteriorated and died on 27 August 2018 with a clinical picture of refractory failure of multiple organs. The suspicion of West Nile virus infection arose only post mortem, after consultation with an infectiologist; additional serological and molecular examinations then confirmed a WNV infection.

**Epidemiological background:** The first epidemiological inquiry was realized relatively soon after the patient´s death and focused primarily on environmental conditions at the place of residence. There were several barrels and tanks filled with stagnant water at the sites, which served as a suitable attractant for mosquitoes. Based on these findings, several CO_2_ mosquito traps (Bioquip, Rancho Dominguez U.S.) were installed on 2 capture nights. A total of 87 female mosquitoes belonging to *Cx. pipiens*, *Anopheles maculipennis* sensu lato, *Aedes vexans*, *Ae. caspius* and *Ae. sticticus* were captured and examined for the presence of WNV. No positive sample was found. The patient did not leave the residence. Mosquitoes bit her regularly. She was not vaccinated against TBEV and did not undergo blood transfusion.

#### 2.1.3. Case 3

The 51-year-old man was admitted on 2 October 2018 to the Infectious Diseases Department of Břeclav Hospital for non-specific symptoms lasting from 9 September 2018—fever, headaches, fatigue, arthralgia and sleep disorders. This was a patient without significant comorbidities, who in the past was treated for Lyme disease and vertebrogenic algic syndrome. As part of the diagnosis, a lumbar puncture was added; biochemical and cytological tests of cerebrospinal fluid corresponded to aseptic neuroinfection. A CT scan of the brain showed no recent pathological changes. Tick-borne meningoencephalitis and herpesvirus or enterovirus neuroinfections were considered for differential diagnosis, but microbiological tests were negative for all these agents. The infectiologist also indicated that serological diagnosis of West Nile fever will be performed at the National Reference Laboratory (NRL) for arboviruses (Public Health Institute, Ostrava). Additional laboratory tests then confirmed a WNV infection. The clinical course of the disease was mild in this case; there were no complications during hospitalization, and the patient was discharged home after 14 days without a residual neurological deficit.

**Epidemiological background:** The epidemiological examination was performed soon after the patient returned from the hospital. The patient lives in a family house with garden and small natural pond; in some places there are barrels of stagnant water. He did not travel outside his residence, except for regular walks in the nearby forest. He denied vaccination against TBEV as well as blood donation/transfusion.

#### 2.1.4. Case 4

A 52-year-old man was admitted on 18 September 2018 to the Infectious Diseases Department of the Břeclav Hospital for non-specific symptoms lasting 8 days. At first, there were high fever, fatigue, arthralgia and myalgia, and the patient also reported headaches, diarrhea and vomiting during the past 4 days. The patient’s medical history included arterial hypertension and long-term headaches of unclear origin. In the department of infectious diseases, lumbar puncture was added; biochemical and cytological tests of cerebrospinal fluid corresponded to aseptic neuroinfection. Due to the characteristics of the complaint, a CT scan of the brain was added with a finding of incipient cerebral oedema. However, later magnetic resonance imaging (MRI) of the brain did not show any intracranial inflammatory changes. The results of routine serological tests (including serology in tick-borne meningoencephalitis) were not convincing. In the light of previous experience (see Case reports 2 and 3), biological material was sent to the NRL for arboviruses for the diagnosis of West Nile fever. The laboratory also confirmed WNV infection in this case. In this case the hospitalization lasted 17 days, there were no complications, and the patient was discharged home in good condition.

**Epidemiological backgound:** Epidemiological investigation was realized by phone call. Patient lives in a family house with garden on the margin of town, close to a fishpond accompanied with rich vegetation. In the garden, several objects (barrels, watering cans) with stagnant water were present. He did not travel outside the region. He denied vaccination to TBEV as well as blood donation/transfusion.

#### 2.1.5. Case 5

A 46-year-old woman without a serious previous illness was admitted to the Infectious Diseases Clinic of Brno University Hospital on suspicion of meningoencephalitis on 10 January 2018. A 10-day tourist stay in Turkey preceded the symptoms, but the development of the symptoms occurred on the 3rd day after leaving for Turkey from the Czech Republic. First, a striking fatigue developed, with the development of headache in the following days and accompanied with nausea and vomiting, vertigo, and later the family observed bradypsichia and dysarthria. She had chills but did not measure her temperature. After admission, the patient was afebrile, stable in terms of the circulatory system, stupor, bradypsichia, still oriented in all qualities, with positive upper meningeal phenomena in the neurostat, fine tremor of the upper extremities and dysmetria, ataxia, standing titubation without lateral predilection. Other objective findings were remarkable (see Table 2). Only a slight increase in CRP was observed in the laboratory analysis; the other biochemical and haematological parameters were without deviations from the standard. On the day of the admission, a sample of cerebrospinal fluid was taken from the patient for examination—a picture of monocytic pleocytosis was present in the cerebrospinal fluid, the diagnosis of serous meningoencephalitis was confirmed in the context of the clinical condition. Anti-oedema therapy was administered to the patient—corticoids and osmotic diuretics—and a strict rest regime was indicated. Cerebrospinal fluid was sent for serological and molecular genetic testing. Herpes infection and enterovirus infections were eliminated by PCR. Tick-borne meningoencephalitis and Lyme borreliosis were also serologically excluded. After excluding other probable causes, the West Nile virus was suspected—cerebrospinal fluid, serum and urine were sent to the NRL for arboviruses for further analysis. The patient’s health gradually improved during hospitalization, and anti-oedema doses decreased. As a complication of the corticosteroid therapy, the patient developed deep vein thrombosis of the right lower limb; given this result, anti-coagulant therapy was indicated. The complete serological results of the NRL examination were not absolutely unambiguous; admitted as a possible WNV pathogen, but Usutu virus infection was considered more likely. There is no causal treatment for any of these diseases, only symptomatic treatment is available; the patient was discharged from the hospital in good clinical condition on the 12th day of the hospitalization. At the follow-up visit on day 10 after discharge, the patient was completely free of symptoms; the neurological finding was negative.

**Epidemiological background**: Due to the development of symptoms soon after arrival in Turkey, the patient probably developed the disease before departure. The patient has a permanent residence in South Moravia and has reported mosquito biting.

### 2.2. Diagnostic Summary 

Diagnostic outcomes are summarized in Table 3. The diagnosis of our patients was based on a case definition published by European Union [EU]. RealStar^®^ WNV RT-PCR Kit 1.0 (Altona Diagnostics GmbH, Hamburg, Germany) assay was used for PCR and positive samples were sequenced [15]. Patient sera were tested for anti-WNV IgG and IgM with three commercial assays: Anti-West Nile virus IIFT IgG and IgM, Anti-West Nile virus ELISA IgG and IgM (both Euroimmun, Lübeck, Germany), ELISA West Nile Virus IgG and IgM capture Dx SelectTM (Focus Diagnostics, Cypress, CA, USA) and for anti-TBEV IgG and IgM with ELISA Viditest anti-TBEV IgG, IgG avidity and IgM (Vidia, Vestec, Czech Republic). VNT for WNV, USUV and TBEV according to a previously published internal protocol were used to confirm serological results [16,17].

In three patients both PCR and serology were positive, while one was not tested for PCR and one had positive antibodies but negative PCR. Only one of the WNV-positive samples (Patient 2) was confirmed to be a WNV-lineage 2 virus (by sequencing) and revealed a WNV-2 strain almost identical to WNV-2, which was detected in local mosquitoes [3]. Other samples could not be sequenced due to a low amount of viral RNA in samples. Therefore, USUV identification was not confirmed by sequencing or PCR. Positive serology was confirmed by a virus neutralization test (VNT) for WNV in all 5 patients; therefore, they met the laboratory criteria for a confirmed case. Subsequently, established VNT for USUV detected anti-USUV antibodies in 4 of the 5 previously diagnosed WNV patients. Three patients had anti-USUV VNT titers significantly lower than anti-WNV, presumably corresponding to cross-reactivity. The 5th patient had anti-USUV VNT titer 16 times higher than the anti-WNV, implying she was most likely to have a USUV infection, although her results originally matched the EU definition for WNV. Regarding serology, cross-reactivity was detected in the ELISA, while no patient had a equivocal positive anti-TBEV VNT.

## 3. Discussion

West Nile fever is now the most important mosquito-borne viral disease in Europe. The incidence of WNV peaked in 2018 with a total number of 2083 confirmed human cases (a 7.2-fold increase over the previous year) in Europe [18]. The massive outbreaks affected mainly Southern (Italy, Greece, Spain, France), Eastern (Croatia, Serbia, Bulgaria, Romania) and Central (Austria, Hungary, the Czech Republic) Europe, and expansion into previously virus-free regions (Slovenia, Kosovo). In a broader context, we should also consider the role of genetic, ecological, environmental and possible socio-economic aspects that may have played a role in increased WNV activity during the 2018 transmission season, most importantly suitable environmental factors for mosquito vectors, particularly increased day temperature [18] as well as more specific environmental factors such as a large number of vessels with stagnant water (barrels, watering cans and containers), which are constantly found in urban areas in the summer months (applies mainly to the Czech Republic). These objects represent ideal places for mass breeding of WNV vectors.

The Czech Republic and Germany are countries with the northernmost spread of WNV in Europe [3,19]. Increased surveillance, including large-scale surveys of mosquitoes, horses and birds, carried out during two large-scale EC-funded cooperation projects (EDEN and EDENext) between 2008 and 2015, has long indicated that WNV cases may occur in the country. As for the supervision of birds, Hubálek et al. [20] examined 54 domestic birds (geese and ducks) and 391 wild birds representing 28 migratory and resident species, using VNT in the South Moravian fishpond ecosystem. Antibodies to WNV were not detected in domestic waterfowl, but 23 (5.9%) wild birds of 10 species showed a positive response. Straková et al. [21] examined antibodies against WNV and USUV in 146 common coots (*Fulica atra*) on ponds in Moravia. Our results show that both WNV and USUV infections occur in common coots, and this species of bird can serve as an “indicator” of the presence of these viruses in fishpond and wetlands in Central Europe. In addition, two goshawks (*Accipiter gentilis*) held captive by falconers in Moravia died of WNV encephalitis in 2017 [22] and among predators, especially goshawks (several of them wild), WNV encephalitis broke out in the Czech Republic in 2018 [23].

According to recent data from several European countries [24,25], goshawks can serve as suitable indicators for active WNV circulation during the summer season in Europe. As far as horse surveillance is concerned, no case of West Nile fever has been reported in horses so far. The State Veterinary Institute in cooperation with the reference laboratory for arboviruses, regularly examines horse sera from all districts in the Czech Republic. Blood sera from 163 horses were examined from various parts of the Czech Republic in a plaque reduction neutralization test (VNT), but no specific WNV antibodies were detected [26]. A similar examination of a much larger sample of horses (2349 animals) revealed 11 horses (0.47%) with specific antibodies to WNV [27]. Regarding mosquito monitoring, WNV-2 was detected (RT-PCR) in *Culex modestus* mosquitoes collected in ponds in South Moravia during August 2013 and also isolated (newborn mice). Phylogenetic analysis has shown that these Czech WNV strains are closely related to the Austrian, Italian and Serbian strains reported in 2008, 2011 and 2012, respectively [3]. A total of 61,770 female *Cx. modestus* were collected in South Moravian ponds in the years 2010 to 2014, and 1243 samples were examined for the presence of flaviviruses by RT-PCR. Nine strains of WNV lineage 2 were detected in *Cx. modestus* collected in the same reed ecosystem. USUV and WNV co-circulate in the same wetland ecosystem, characterized by the presence of waterfowl and *Cx. modestus* mosquitoes, serving as hosts and vectors, respectively, for both viruses [8]. In addition, ornithophilic *Cx. pipiens* was demonstrated as a vector in 2015 [4]. A total of 28,287 hibernating mosquitoes caught in February or March from 2011 to 2017 in a WNV-endemic area of South Moravia were screened for the presence of WNV RNA. No WNV positive pools were found from 2011 to 2016, while lineage 2 WNV RNA was detected in 3 pools of *Cx. pipiens* mosquitoes collected in 2017 at 2 study sites. The data support the hypothesis of possible WNV persistence in mosquitoes throughout the winter season in Europe [28]. Interestingly, antibodies to WNV (overall 5.9% prevalence) were documented by VNT in the blood sera of wild artiodactyls including roe deer, red deer, fallow deer, mouflons and wild boars, sampled in the South Moravian district of Břeclav [29].

Blood safety testing started after the first WNV human cases were confirmed in the affected area (from September until November 2018). The Transfusion and Tissue Department of the University Hospital Brno started WNV testing of blood donors by PCR in September 2018 and finished at the end of November 2018. This solution was based on an epidemiological situation in South Moravia published on the websites of the European Centre for Disease Prevention and Control (ECDC). During this period, 4400 blood donors were tested with negative results.

## 4. Conclusions

In conclusion, our results confirm simultaneous circulation of WNV and USUV in the Czech Republic, so far limited to Southern Moravia. However, the first WNV-positive mosquitoes recently found in another region of the Czech Republic (Southern Bohemia) may indicate new WNV focus in the country [30]. Only a One-Health approach practicing interdisciplinary collaboration among local infection specialists, epidemiologists, veterinarians and entomologists, can bring benefit in the prevention and control of WNV in affected areas and also detect the introduction of WNV in previously virus-free areas in a timely manner.

## Figures and Tables

**Table 1:** Descriptive epidemiological and clinical summary of five patients diagnosed with mosquito-borne disease, from July to October 2018, Czech Republic.

	Case 1	Case 2	Case 3	Case 4	Case 5
**Demographic data**					
Age (years)	74	72	51	52	46
Gender	Male	Female	Male	Male	Female
**Epidemiological data**					
Area of residence	Urban	Urban	Urban	Urban	Urban
Occupation	Retired	Retired	Construction Manager	Sales Representative	Shop Assistant
Travel history	Austria (1 day)	No	No	No	Turkey
Outdoor activity	fishing	gardening	walking	gardening	gardening
Contact with mosquitoes	Yes	Yes	Yes	Yes	Yes
Vaccination status (TBE, YF)	Yes (TBE)	No	No	No	No
Blood donation/Transfusion	No	No	No	No	No
**Clinical presentation**					
Date of disease onset	23 July 2018	7 August 2018	10 September 2018	11 September 2018	22 September 2018
Days of hospitalization	N/A	15	14	17	12
Main clinical signs and symptoms	Fever, headache, muscle pain, diarrhea, macular exanthema	Fever, diarrhea, muscle pain, weakness	Fever, headache, arthralgia, fatigue	Fever, fatigue, diarrhea, arthralgia, headache, vomiting	Fever, headache, vomiting, ataxia, meningeal signs
The highest body temperature	40 °C	39 °C (anamnestic)	37.6 °C	39.4 °C	Not measured at home
The lowest GCS	15	3	15	15	14
Comorbidities	Ischemic heart disease, arterial hypertension, HLA-B27 positive	Rheumatoid arthritis, Lichen ruber, chronic gastritis, hypothyroidism	Vertebrogenic algic syndrome	Chronic cefalea, hypertension	Gastroesophageal reflux disease
Clinical diagnosis	Fever	Meningoencephalitis	Meningitis	Meningitis	Meningitis
Duration of disease (No. days)	13	20	36	24	22
Outcome at discharge (GOS)	Recovered (8)	Deceased	Recovered (8)	Recovered (8)	Recovered (8)

Legend: TBE-Tick-borne encephalitis; YF-Yellow fever; GCS-Glasgow Coma Scale; GOS-Glasgow Outcome Scale; NA—not applicable.

**Table 2:** Laboratory and neuropathological findings of five patients diagnosed with mosquito-borne disease, from July to October 2018, Czech Republic.

	Case 1	Case 2	Case 3	Case 4	Case 5	Reference Range
**Cerebrospinal fluid (CSF) examination**	Day 9	Day 13	Day 22	Day 8	Day 10	
Cell count/mm^3^	-	5	12	15	115	0–5
Polymorphonuclear/mononuclear cells	-	-	-	-	5/110	-
Proteins (g/L)	-	0.6	-	0.99	0.87	0.15–0.45
Glucose (mmol/L)	-	4.3	-	4.3	2.9	N/A
Lactate (mmol/L)	-	-	-	2.9	2.7	1.1–2.4
**Serum examination**						
C-reactive protein; CRP (mg/L)	1.4	25	12	5	22.7	0-5
White blood cells; WBC (×10^9^/L)	8.54	16.22	7.19	8.05	5.19	4–10
Platelets (×10^9^/L)	243	310	225	118	244	150–400
Red blood cells; RBC (×10^12^/L)	4.88	5.24	5.29	4.44	4.08	4.0–5.8
Hemoglobin (g/L)	152	154	150	138	125	135–175
Bilirubin (μmol/L)	11.5	4.9	-	21.0	7.6	2–21
Aspartate-aminotransferase; AST (μkat/L)	0.48	0.77	-	0.82	0.35	0.17–0.85
Alanine-aminotransferase; ALT (μkat/L)	0.51	0.81	-	2.25	0.28	0.17–0.83
Gamma-glutamyltransferase; GGT (μkat/L)	1.20	-	-	-	0.69	0.13–1.02
Lactate dehydrogenase; LD (μkat/L)	4.08	-	-	-	N/A	2.25–3.75
**Brain examination/imaging**						
Brain computed tomography; CT	-	hypodensity vs.postinfectious	no pathological findings	onset of oedema	-	-
Brain magnetic resonance imaging; MRI	-	-	-	without signs of inflammation	-	-

**Table 3:** Diagnostic outcomes of five patients diagnosed with mosquito-borne disease, from July to October 2018, Czech Republic.

Case	Days Tested	WNV PCR (S,WB, CSF,U)	IgM ^1^ WNV ELISA Euroimmun ^2^ WNV ELISA Focus ^3^ WNV IIFT Euroimmun	IgG ^1^ WNV ELISA Euroimmun ^2^ WNV ELISA Focus ^3^ WNV IIFT Euroimmun	WNV VNT (titre)	^4^ TBEV IgM ELISA (IP)	^4^ TBEV IgG ELISA (IP)	^4^ TBEV IgG avidity (%)	TBEV VNT (Titre)	USUV VNT (Titre)	Case Status
1	23.10. 2018		0.91 (eq.) ^1^ **1.57 (pos.)** ^2^	**3.49 (pos)** ^1^	**512 (pos.)**	neg.	**12.67 (pos.)**	100%	32 (pos.)	**128 (pos.)**	WNV confirmed
2	18.8. 2018		>**20 (pos.)** ^3^	>**20 (pos.)** ^3^	4 (eq.)	neg.	neg.		neg.		WNV confirmed
	20.8. 2018	S-inhib. **CSF-pos.**	>**20 (pos.)** ^3^	>**20 (pos.)** ^3^	**8 (pos.)**						
	24.8. 2018	**S-pos.** CSF-neg.	>**20 (pos.)** ^3^	>**20 (pos.)** ^3^	**16 (pos.)**						
	28.8. 2018	**U-pos.**									
3	3.10. 2018	S,CSF,U-neg.	**3.11 (pos.)** ^1^ **7.22 (pos.)** ^2^ >**20 (pos.)** ^3^	1.10 (eq.) ^1^ 1.02 (eq.) ^2^ >**20 (pos.)** ^3^	64 (pos.)	neg.	neg.		neg.	**16 (pos.)**	WNV confirmed
	4.10. 2018	**WB-pos.**									
	10.10. 2018	**WB-pos.** S,U-neg.	**5.63 (pos.)** ^2^	**2.28 (pos.)** ^2^	**128 (pos.)**	neg.	neg.		neg.	**32 (pos.)**	
	8.11. 2018	**WB-pos.** S,U-neg.	**1.65 (pos.)** ^1^	**2.82 (pos.)** ^1^	**32 (pos.)**	neg.	**1.81 (pos.)**	33 %	neg.	**8 (pos.)**	
	13.12. 2018	**WB-pos.** U-neg.	**1.61(pos.)** ^1^	**3.13 (pos.)** ^1^	**32 (pos.)**	neg.	**2.41 (pos.)**	36 %	neg.	neg.	
	15.2. 2019	**WB-pos.**;U-neg.									
4	18.9. 2018	S-neg.	**2.36 (pos.)** ^1^ **5.27 (pos.)** ^2^ >**500 (pos.)** ^3^	neg. ^1^ neg. ^2^ **20 (pos.)** ^3^	**neg.**	neg.	neg.		neg.	neg.	WNV confirmed
	24.9. 2018	S-neg. **U-pos.**	**2.90 (pos.)** ^1^ **6.39 (pos.)** ^2^ **500 (pos.)** ^3^	**1.31 (pos.)** ^1^ **1.26 (pos.)** ^2^ >**20 (pos.)** ^3^	**8 (pos.)**	0.96 (eq.)	**1.19 (pos.)**	38 %	neg.	4 (eq.)	
	1.10. 2018	S, U-neg.	**3.69 (pos.)** ^1^ **6.28 (pos.)** ^2^	**2.12 (pos.)** ^1^ **2.13 (pos.)** ^2^	**64 (pos.)**	neg.	**2.18 (pos.)**	22 %	neg.	**16 (pos.)**	
	4.10. 2018	**WB-pos.**									
	19.10. 2018	WB, U-neg.	**3.10 (pos.)** ^1^	**3.16 (pos.)** ^1^	**64 (pos.)**	neg.	**2.75 (pos.)**	20 %	neg.	4 (eq.)	
	25.1. 2019	WB neg. **U-pos.**	neg. ^1^	**3.05 (pos.)** ^1^	**32 (pos.)**	neg.	**2.10 (pos.)**	43 %	neg.	neg.	
5	1.10. 2018	S, CSF-neg.	neg. ^1,2,3^	neg. ^1,2,3^	neg.	neg.	neg.		neg.	**16 (pos.)**	USUV
	5.10. 2018	WB, U-neg.	neg. ^1,2^ >**20 (pos.)** ^3^	neg. ^1,2,3^							
	10.10. 2018		**1.37 (pos.)** ^1^ **20 (pos.)** ^3^	neg. ^1^ **20 (pos.)** ^3^	**16 (pos.)**	neg.	neg.		neg.	**256 (pos.)**	
	25.10. 2018		neg. ^2^	0.93 (eq.) ^1^							

Legend: S-serum, WB-whole blood, CSF-cerebrospinal fluid, U-urine, IP-positivity index, IIFT-indirect immunofluorescence, VNT-virus neutralization test, pos.-positive, neg.-negative, eq.-equivocal, inhib.-inhibition; Reference values for WNV ELISA Euroimmun IgG and IgM (IP): <0.80 negative, 0.80–1.10 equivocal, >1.10 positive; Reference values for WNV ELISA Focus IgG (IP) <1.30 negative, 1.30–1.50 equivocal, >1.50 positive; Reference values for WNV ELISA Focus IgM (IP) <0.90 negative, 0.90–1.10 equivocal, >1.10 positive; Reference values for TBEV ELISA IgG and IgM (IP): <0.90 negative, 0.90–1.10 equivocal, >1.10 positive; Reference values for TBEV ELISA IgG avidity (%): <40 % low, 40–60 equivocal, >60 high; Reference values for WNV IIFT Euroimmun: <20 negative, ≥20 positive; Reference values for VNT( titre): <4 negative, 4 equivocal, >4 positive. All positive values are highlighted in bold. ^1^ West Nile Virus ELISA IgG, IgM ELISA, Euroimmun, Lübeck, Germany (Cat Nr. EI 2662-9601 G,M); ^2^ West Nile Virus ELISA IgG, IgM capture Dx SelectTM ELISA, Focus Diagnostics, Cypress, California, U.S. (Cat Nr EL 0300 G,M); ^3^ West Nile Virus IgG, IgM IIFT, Euroimmun, Lübeck, Germany (Cat Nr. FI 2662-1005 G,M); ^4^ ELISA-Viditest anti-TBEV IgG, IgG avidity and IgM, Vidia, Vestec, Czech Republic (Cat Nr. OD-170, OD-194).

## Data Availability

Not applicable.
